# Prediction of non-responsiveness to pre-dialysis care program in patients with chronic kidney disease: a retrospective cohort analysis

**DOI:** 10.1038/s41598-021-93254-0

**Published:** 2021-07-06

**Authors:** Emily K. King, Ming-Han Hsieh, David R. Chang, Cheng-Ting Lu, I-Wen Ting, Charles C. N. Wang, Pei-Shan Chen, Hung-Chieh Yeh, Hsiu-Yin Chiang, Chin-Chi Kuo

**Affiliations:** 1 Department of Medical Media Design and Application, Interpedia Incorporated, Taichung, Taiwan; 2 Division of Nephrology, Department of Internal Medicine, China Medical University Hospital and College of Medicine, China Medical University, Taichung, Taiwan; 3 Big Data Center, China Medical University Hospital and College of Medicine, China Medical University, 2, Yude Rd., North Dist., Taichung City, 404 Taiwan; 4 Department of Bioinformatics and Medical Engineering, Asia University, Taichung, Taiwan; 5 Center for Artificial Intelligence and Precision Medicine Research, Asia University, Taichung, Taiwan; 6 AKI-CARE (Clinical Advancement, Research and Education) Center, Department of Internal Medicine, China Medical University Hospital and College of Medicine, China Medical University, Taichung, Taiwan

**Keywords:** Diseases, Medical research, Nephrology

## Abstract

The responsiveness of patients with chronic kidney disease (CKD) to nephrologists’ care is unpredictable. We defined the longitudinal stages (LSs) 1–5 of estimated glomerular filtration rate (eGFR) by group-based trajectory modeling for repeated eGFR measurements of 7135 patients with CKD aged 20–90 years from a 13-year pre-end-stage renal disease (ESRD) care registry. Patients were considered nonresponsive to the pre-dialysis care if they had a more advanced eGFR LS compared with the baseline. Conversely, those with improved or stable eGFR LS were considered responsive. The proportion of patients with CKD stage progression increased with the increase in the baseline CKD stage (stages 1–2: 29.2%; stage 4: 45.8%). The adjusted times to ESRD and all-cause mortality in patients with eGFR LS-5 were 92% (95% confidence interval [CI] 86–96%) and 57% (95% CI 48–65%) shorter, respectively, than in patients with eGFR LS-3A. Among patients with baseline CKD stages 3 and 4, the adjusted times to ESRD and all-cause death in the nonresponsive patients were 39% (95% CI 33–44%) and 20% (95% CI 14–26%) shorter, respectively, than in the responsive patients. Our proposed Renal Care Responsiveness Prediction (RCRP) model performed significantly better than the conventional Kidney Failure Risk Equation in discrimination, calibration, and net benefit according to decision curve analysis. Non-responsiveness to nephrologists’ care is associated with rapid progression to ESRD and all-cause mortality. The RCRP model improves early identification of responsiveness based on variables collected during enrollment in a pre-ESRD program. Urgent attention should be given to characterize the underlying heterogeneous responsiveness to pre-dialysis care.

## Introduction

Delaying the progression of chronic kidney disease (CKD) to end-stage renal disease (ESRD), which requires renal replacement therapy, is a theoretically possible strategy for improving patient outcomes and radically reducing healthcare costs, particularly in countries with endemic ESRD. Identifying patients with CKD who respond and do not respond to nephrologists’ care is crucial but challenging, even in a managed care model, such as the multidisciplinary pre-dialysis care, first advocated in 1994 by the US National Institutes of Health^1–3^. Indeed, recent studies have shown inconsistent results regarding the potential of intense pre-dialysis care in slowing or arresting CKD progression^4–11^. For example, a recent study showed that among older veterans, the lower intensity of pre-dialysis management in the Veterans Affairs health care system than in Medicare was not associated with higher mortality^11^. Two studies that used a propensity score matched case–control design revealed a high dialysis rate among patients with pre-dialysis care during the progression of CKD to ESRD; however, neither study considered the potential competing risk of death^5,6^. By contrast, most studies based on the national pay-for-performance pre-dialysis care program showed overall improvements in mortality, dialysis rate, mature vascular access, and general cost saving^8–10^.

Prior studies have examined the longitudinal trajectories of estimated glomerular filtration rate (eGFR) in multiple CKD populations. Li et al. identified four main eGFR progression features based on an African American Study of Kidney Disease and Hypertension consisting of 846 patients. An important feature captured was the “fast-decline” trajectory, with a decline rate of at least 4 mL/min/1.73 m^2^/y. Similarly, the “fast-decline” characteristic of longitudinal eGFR trajectory is consistently recognized in diverse populations with CKD or diabetes^12–15^. However, whether this “fast-decline” trajectory can be mitigated through nephrologist-driven pre-dialysis care remains undetermined. In the present study, by using a national registry-based pre-ESRD cohort with a standardized care protocol, we defined the longitudinal CKD stage through modeling trajectories of all eGFR measurements following enrollment in the pre-dialysis program and defined the individual’s response to pre-dialysis care based on the discrepancy between baseline CKD stage at enrollment and the longitudinal CKD stage. We aimed to develope a prediction model to help early identification of non-responsiveness to pre-dialysis care.

## Methods

### Study population

Taiwan’s National Health Insurance launched the Project of Integrated Care of CKD in 2002, initially targeting patients with an eGFR of < 60 mL/min/1.73 m^2^ or proteinuria (urine protein-to-creatinine ratio [uPCR] > 1 g/g creatinine). In 2007, the program used a multidisciplinary approach to focus on CKD stages 3b–5^4,16^. China Medical University Hospital (CMUH), a tertiary medical center in Central Taiwan, joined the program in 2003 and prospectively enrolled consecutive patients with CKD who were willing to participate^17^. Details of the program were described in a previous study^18^. Biochemical markers of renal injury, including serum creatinine (S-Cre), blood urea nitrogen, and spot uPCR, were measured at least once every 3 months. We further integrated the CMUH pre-ESRD program with the CMUH electronic medical records (EMRs) containing laboratory tests, medications, special procedures, and admission records^19^. All enrolled patients were followed-up until initiation of long-term renal replacement therapy (hemodialysis, peritoneal dialysis, or transplantation), loss to follow-up, death, or December 31, 2016, whichever occurred first.

In the present study, we included participants of the pre-ESRD program from Jan 2003 to Dec 2015 who were aged 20–89 years, had no history of dialysis, and had at least 2 measurements of serum S-Cre during the follow-up period. Then, we excluded participants with a time between baseline and final S-Cre measurements of < 6 months. In total, 7135 participants with 156,295 records of S-Cre were included in the analysis (Figure S1). The study was approved by the Big Data Center of CMUH and the Research Ethical Committee/Institutional Review Board (REC/IRB) of China Medical University and informed consent was waived (CMUH105-REC3-068). All methods were performed in accordance with the relevant guidelines and regulations of REC/IRB.

### Determination of kidney function

S-Cre levels were measured using the Jaffe rate method (kinetic alkaline picrate) at CMUH Central Laboratory by using a Beckman UniCel DxC 800 immunoassay system (Beckman Coulter Inc., Brea, CA, USA). The eGFR was calculated using the Chronic Kidney Disease Epidemiology Collaboration creatinine equation^20^. The S-Cre level at enrollment was used to define the baseline eGFR and the corresponding CKD stages according to the following cutoff values: > 90 [stage 1], 60–89.9 [stage 2], 45–59.9 [stage 3a], 30–44.9 [stage 3b], 15–29.9 [stage 4], and < 15 [stage 5] mL/min/1.73 m^2^. All S-Cre measurements of the enrolled participants were considered until the study endpoints. The quarterly average eGFR level was calculated if the patient had received more than one eGFR measurement in a 3-month period, and the individual’s eGFR trajectory was modeled based on quarterly average eGFR measures. For patient with only urine albumin-to-creatinine ratio (uACR) available, we converted uACR into uPCR based on the following equation derived from a Japanese study: $\ln \left( {{\text{ACR}}} \right) = 1.32*\ln \left( {{\text{PCR}}} \right) - 2.64$^21^. Proteinuria was defined as uPCR > 0.5 g/g creatinine. Details of other co-variables are summarized in the Supplementary Material.

### Longitudinal stage of eGFR and Responsiveness to pre-dialysis care

Longitudinal stage of eGFR (eGFR LS) was determined based on the eGFR trajectories derived from group-based trajectory modeling (GBTM), which is described detail in the section of statistical analyses. Patients were considered nonresponsive to the pre-ESRD program if they reached a more advanced longitudinal stage of eGFR than the baseline CKD stage. Conversely, those with improved or stable CKD stages were defined as responsive patients.

### Statistical analyses

Continuous variables were compared using the Wilcoxon rank sum test and expressed as median and interquartile range (IQR), whereas categorical variables were compared using the chi-square test and expressed as frequency (percentage). We used semiparametric GBTM to characterize the follow-up period trajectories of all eGFR measurements of the patients enrolled in the CMUH pre-ESRD program. Briefly, the PROC TRAJ macro developed using the SAS software was used to fit a semiparametric mixture model to the longitudinal data by using the maximum likelihood method^22–24^. This approach is useful when the number of subgroups and other information, such as the trajectory shapes of each subgroup, are unknown. We empirically compared 3-, 4-, 5-, and 6-group solutions and then optimized the number of subgroups by using Bayesian information criterion values (close to zero indicated a good fit); the trajectory shapes were determined according to the order of the polynomial (e.g., linear, quadratic, and cubic). The eGFR trajectories were determined before analysis of the risk of dialysis and mortality.

To construct time-to-event analysis, person-years free of dialysis and mortality after the pre-ESRD enrollment were computed along with Kaplan–Meier survival functions. We evaluated the prospective associations of longitudinal eGFR trajectories and responsiveness to the pre-dialysis care with the risk of dialysis initiation and mortality by using multiple Cox proportional hazards models. The models were adjusted progressively (see footnotes of Table 3). To characterize the dialysis risk associated with exposures of interest, we performed a competing risk analysis according to the protocol provided by Fine and Gray, which minimized the potential bias introduced by a competing death risk^25^. Furthermore, we performed a parametric survival analysis under the Weibull distributions and applied the same adjustment strategy as in the Cox modeling. The relative survival time was estimated by exponentiating the coefficient of the main predictors (eGFR trajectories or responsiveness to pre-dialysis care). The relative time can be interpreted as the difference between the time required for individuals in the exposed population and nonexposed population to experience the events of interest. The model fitting was evaluated by using the Akaike information criterion. Due to missing data on some explanatory variables (e.g., urinary protein-to-creatinine ratio (uPCR) up to 28%; Table S1), we further performed multiple imputations with a fully conditional method in SAS, namely an iterative Markov chain Monte Carlo procedure, to replace the missing values for uPCR, comorbidities, and medications with imputed values. We specified the number of imputations as 20 and the number of iterations as 100. Exploratory subgroup analyses were performed to evaluate potential effect modification among patients with different longitudinal eGFR trajectories. To explore potential effect modifier in the association between responsiveness and main outcomes, we stratified patients on the basis of age older or younger than 65 years, sex, BMI category (< 24, 24–47, > 27 kg/m^2^), smoking status, CKD stage (3 vs. 4 or 5), uPCR higher or lower than 500 mg/g creatinine, serum uric acid (SUA) higher or lower than 7 mg/dL, diabetes, hypertension, and CVD at baseline. To evaluate the responsiveness prediction model, we compared the C-statistic between the reference Kidney Failure Risk Equation composed of age, sex, eGFR, uPCR, serum calcium, phosphorus, and albumin and our proposed model, Renal Care Responsiveness Prediction (RCRP), which additionally used DM, HTN, hemoglobin, and the history of NSAID exposure. We also plotted the observed versus predicted risk probability to reveal the differences in calibrations of all the responsiveness-prediction models. A decision curve analysis was then conducted to determine the clinical usefulness of the proposed prediction models quantifying the net benefit at different threshold probabilities^26,27^. All statistical analyses were performed using SAS version 9.4 (SAS Institute Inc., Cary, NC, USA) and R version 3.5.1 (R Foundation for Statistical Computing, Vienna, Austria). The two-tailed statistical significance level of α was set at 0.05.

### Ethical approval

The study was approved by the Research Ethical Committee/Institutional Review Board of China Medical University Hospital (CMUH105-REC3-068).

## Results

### Clinical characteristics across prospective eGFR longitudinal stage

**Figure 1:**
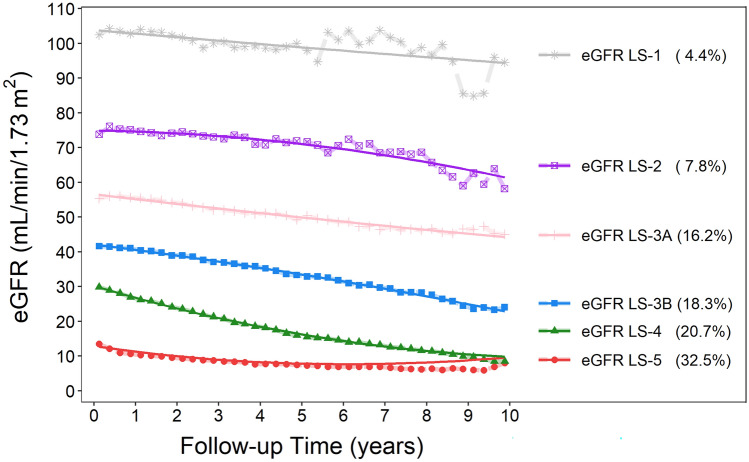
Longitudinal stages of eGFR trajectory defined by using GBTM according to serial quarterly average levels of eGFR over the CKD course. The solid line is the averaged estimated trajectory, and the points represent the averaged observed trajectory.

The median (IQR) age at enrollment of all 7135 participants was 67.5 years (IQR 57.1–76.3); median follow-up duration was 2.40 and 4.25 years for ESRD events and death, respectively; and median (IQR) frequency of eGFR measurements was 16 times (IQR 9–30). We identified 6 eGFR trajectories by using GBTM that correspond well to the eGFR range of the current CKD stages of 1, 2, 3a, 3b, 4, and 5. (Fig. 1). We then considered eGFR LS-1 and LS-2 as the reference group. Compared with the reference group, those in the longitudinal eGFR LS-3B and LS-4 were considerable older and with a higher prevalence of CVD at baseline (Table 1). A decreasing trend of baseline BMI for increasing eGFR LSs was observed, which was opposite to the trend of the baseline prevalence of diabetes and hypertension. At baseline, nonsteroidal anti-inflammatory drug (NSAID) exposure was relatively prevalent, ranging from 21.9 for eGFR LS-5 to 28.6% for eGFR LS-3A. For exposure to radiocontrast, it ranged from 6.2 for eGFR LS-1-2 to 11.5% for eGFR LS-4 (Table 1).

**Table 1:** Baseline demographic and clinical characteristics according to eGFR LS defined by using GBTM.

Variables	Total (N = 7135)	eGFR LS-1 or 2 (n = 876)	eGFR LS-3A (n = 1153)	eGFR LS-3B (n = 1308)	eGFR LS-4 (n = 1477)	eGFR LS-5 (n = 2321)	*P *value^†^	*P* for trend^‡^
Age at entry (year), median (IQR)	67.5 (57.1, 76.3)	56.1 (46.7, 65.7)	66.5 (57.5, 75.6)	71.2 (61.9, 78.2)	71.6 (62.0, 78.8)	67.1 (57.1, 75.7)	< 0.001	< 0.001
Female, n (%)	3034 (42.5)	410 (46.8)	322 (27.9)	453 (34.6)	622 (42.1)	1227 (52.9)	< 0.001	< 0.001
BMI (kg/m^2^), median (IQR)	24.3 (22.1, 27.1)	25.1 (22.5, 28.2)	24.8 (22.8, 27.3)	24.7 (22.3, 27.1)	24.3 (22.2, 27.0)	23.8 (21.5, 26.4)	< 0.001	< 0.001
Initial CKD stage, n (%)								
1	297 (4.2)	277 (31.7)	14 (1.2)	5 (0.4)	1 (0.1)	0 (0.0)	< 0.001	–
2	532 (7.5)	334 (38.2)	154 (13.4)	25 (1.9)	18 (1.2)	1 (0.0)		
3	3129 (43.9)	245 (28.0)	945 (82.0)	1139 (87.2)	705 (47.8)	95 (4.1)		
4	1840 (25.8)	17 (1.9)	36 (3.1)	128 (9.8)	717 (48.6)	942 (40.7)		
5	1325 (18.6)	2 (0.2)	4 (0.4)	10 (0.8)	34 (2.3)	1275 (55.1)		
Smoking, n (%)								
Never	5911 (82.9)	703 (80.3)	930 (80.7)	1057 (80.8)	1235 (83.6)	1986 (85.6)	< 0.001	–
Former	537 (7.5)	63 (7.2)	110 (9.5)	121 (9.3)	112 (7.6)	131 (5.6)		
Current	687 (9.6)	110 (12.6)	113 (9.8)	130 (9.9)	130 (8.8)	204 (8.8)		
Alcohol consumption, n (%)								
Never	6519 (91.4)	805 (91.9)	1028 (89.2)	1169 (89.4)	1354 (91.7)	2163 (93.2)	< 0.001	–
Former	366 (5.1)	32 (3.7)	70 (6.1)	80 (6.1)	78 (5.3)	106 (4.6)		
Current	250 (3.5)	39 (4.5)	55 (4.8)	59 (4.5)	45 (3.1)	52 (2.2)		
Education level (year), n (%)								
< 9	1832 (25.7)	172 (19.6)	233 (20.2)	296 (22.6)	433 (29.3)	698 (30.1)	< 0.001	–
9 ≤ ~ < 12	2778 (38.9)	263 (30.0)	434 (37.6)	519 (39.7)	613 (41.5)	949 (40.9)		
12 ≤ ~ < 16	1669 (23.4)	264 (30.1)	308 (26.7)	308 (23.6)	279 (18.9)	510 (22.0)		
16+	856 (12.0)	177 (20.2)	178 (15.4)	185 (14.1)	152 (10.3)	164 (7.1)		
Diabetes, n (%)	2623 (36.8)	308 (35.2)	312 (27.1)	446 (34.2)	607 (41.1)	950 (41.0)	< 0.001	< 0.001
Hypertension, n (%)	4612 (64.7)	496 (56.6)	721 (62.6)	837 (64.1)	1019 (69.0)	1539 (66.5)	< 0.001	< 0.001
Cardiovascular disease, n (%)	2675 (37.5)	189 (21.6)	431 (37.4)	592 (45.3)	624 (42.3)	839 (36.2)	< 0.001	< 0.001
**Baseline medication profiles, n (%)**								
Pentoxifylline	1750 (25.1)	142 (16.8)	211 (18.9)	352 (27.5)	451 (31.1)	594 (26.1)	< 0.001	< 0.001
NSAIDs	1828 (26.2)	229 (27.1)	320 (28.6)	381 (29.7)	403 (27.8)	495 (21.8)	< 0.001	< 0.001
Contrast media	648 (9.3)	52 (6.2)	119 (10.6)	128 (10.0)	167 (11.5)	182 (8.0)	< 0.001	0.750
*Anti-platelet*	2418 (34.7)	191 (22.6)	386 (34.5)	497 (38.8)	595 (41.0)	749 (32.9)	< 0.001	< 0.001
Aspirin	1956 (28.1)	159 (18.8)	326 (29.2)	423 (33.0)	467 (32.2)	581 (25.5)	< 0.001	0.094
Dipyridamole	458 (6.6)	34 (4.0)	59 (5.3)	75 (5.9)	131 (9.0)	159 (7.0)	< 0.001	0.0001
Ticlopidine, Clopidogrel	2126 (30.5)	166 (19.7)	346 (31.0)	460 (35.9)	516 (35.5)	638 (28.0)	< 0.001	0.010
*Urate-lowering agents*	1835 (26.3)	121 (14.3)	299 (26.7)	403 (31.4)	434 (29.9)	578 (25.4)	< 0.001	< 0.001
Allopurinol	800 (11.5)	42 (5.0)	97 (8.7)	147 (11.5)	217 (14.9)	297 (13.1)	< 0.001	< 0.001
Febuxostat	152 (2.2)	9 (1.1)	14 (1.3)	34 (2.7)	30 (2.1)	65 (2.9)	0.003	0.001
Benzbromarone	787 (11.3)	63 (7.5)	165 (14.8)	209 (16.3)	170 (11.7)	180 (7.9)	< 0.001	0.001
Colchicine	825 (11.8)	52 (6.2)	125 (11.2)	176 (13.7)	194 (13.4)	278 (12.2)	< 0.001	< 0.001
Sulfinpyrazone	77 (1.1)	6 (0.7)	17 (1.5)	15 (1.2)	15 (1.0)	24 (1.1)	0.535	0.914
*Anti-hypertension agents*	5682 (81.5)	667 (79.0)	870 (77.8)	1014 (79.1)	1230 (84.7)	1901 (83.5)	< 0.001	< 0.001
ACEI	1454 (20.9)	170 (20.1)	203 (18.2)	231 (18.0)	334 (23.0)	516 (22.7)	0.001	0.001
ARBs	3068 (44.0)	345 (40.9)	475 (42.5)	567 (44.2)	711 (49.0)	970 (42.6)	< 0.001	0.174
Trichlormethiazide	688 (9.9)	69 (8.2)	81 (7.3)	125 (9.8)	164 (11.3)	249 (10.9)	0.001	< 0.001
Furosemide, Spironolactone, Amizide, Indapamide	2954 (42.4)	251 (29.7)	298 (26.7)	478 (37.3)	708 (48.8)	1219 (53.6)	< 0.001	< 0.001
α blocker	1475 (21.2)	89 (10.6)	205 (18.3)	271 (21.1)	331 (22.8)	579 (25.4)	< 0.001	< 0.001
β blocker	2593 (37.2)	235 (27.8)	423 (37.8)	477 (37.2)	565 (38.9)	893 (39.2)	< 0.001	< 0.001
CCB	3365 (48.3)	291 (34.5)	456 (40.8)	548 (42.8)	743 (51.2)	1327 (58.3)	< 0.001	< 0.001
Tolvaptan	1 (0.0)	0 (0.0)	1 (0.1)	0 (0.0)	0 (0.0)	0 (0.0)	0.281	0.296
*Anti-diaetes agents*	2728 (39.1)	312 (37.0)	315 (28.2)	458 (35.7)	633 (43.6)	1010 (44.4)	< 0.001	< 0.001
OAD	2189 (31.4)	293 (34.7)	286 (25.6)	393 (30.7)	516 (35.5)	701 (30.8)	< 0.001	0.438
Insulin	1315 (18.9)	78 (9.2)	86 (7.7)	196 (15.3)	339 (23.4)	616 (27.1)	< 0.001	< 0.001
**Baseline biochemical profiles, median (IQR)**								
eGFR (mL/min/1.73 m^2^)	32.6 (17.5, 50.0)	81.2 (61.8, 98.4)	53.6 (46.4, 58.9)	40.5 (34.6, 46.3)	28.6 (23.4, 34.5)	12.9 (8.9, 18.3)	< 0.001	< 0.001
Serum creatinine (mg/dL)	1.87 (1.36, 3.10)	0.91 (0.72, 1.20)	1.32 (1.16, 1.50)	1.58 (1.39, 1.82)	2.03 (1.70, 2.41)	3.95 (2.95, 5.44)	< 0.001	< 0.001
Blood urea nitrogen (mg/dL)	29.0 (19.0, 46.0)	14.0 (11.0, 19.0)	19.0 (15.0, 23.0)	23.0 (18.0, 28.0)	31.0 (24.0, 39.0)	51.0 (38.0, 66.0)	< 0.001	< 0.001
Serum uric acid (mg/dL)	7.30 (6.10, 8.60)	6.30 (5.20, 7.40)	7.00 (5.70, 8.10)	7.30 (6.10, 8.50)	7.50 (6.40, 8.80)	7.60 (6.40, 8.90)	< 0.001	< 0.001
Sodium (mmol/L)	138 (136, 140)	138 (136, 140)	138 (136, 140)	138 (136, 140)	138 (136, 140)	138 (135, 140)	< 0.001	< 0.001
Potassium (mmol/L)	4.20 (3.80, 4.60)	4.00 (3.70, 4.30)	4.10 (3.80, 4.40)	4.20 (3.80, 4.60)	4.30 (3.90, 4.70)	4.40 (3.90, 4.90)	< 0.001	< 0.001
Calcium (mg/dL))	8.90 (8.50, 9.20)	9.00 (8.50, 9.30)	9.10 (8.70, 9.40)	9.10 (8.80, 9.40)	9.00 (8.60, 9.30)	8.70 (8.30, 9.00)	< 0.001	< 0.001
Phosphate (mg/dL)	4.00 (3.50, 4.60)	3.80 (3.40, 4.30)	3.60 (3.20, 4.10)	3.70 (3.30, 4.20)	3.85 (3.40, 4.30)	4.40 (3.90, 5.20)	< 0.001	< 0.001
Serum Albumin (g/dL)	3.90 (3.50, 4.30)	4.10 (3.60, 4.40)	4.10 (3.90, 4.40)	4.10 (3.70, 4.40)	3.90 (3.50, 4.20)	3.70 (3.20, 4.00)	< 0.001	< 0.001
Hemoglobin (g/dL)	11.2 (9.6, 13.0)	13.3 (12.1, 14.5)	13.1 (11.7, 14.5)	12.4 (10.8, 13.8)	11.2 (10.0, 12.5)	9.7 (8.7, 10.9)	< 0.001	< 0.001
T-CHO (mg/dL)	181 (155, 212)	187 (161, 225)	183 (159, 210)	178 (154, 206)	178 (151, 210)	182 (152, 215)	< 0.001	0.011
TG (mg/dL)	130 (90, 192)	139 (95, 202)	128 (89, 191)	128 (88, 184)	136 (89, 194)	128 (90, 193)	0.161	0.575
Urine creatinine (mg/dL)	84.5 (55.1, 125.9)	105 (61, 160)	111 (69, 157)	98 (62, 139)	86 (56, 122)	67 (48, 92)	< 0.001	< 0.001
Urine PCR (mg/g)	749 (202, 2172)	325 (147, 1000)	165 (86, 435)	272 (124, 791)	687 (253, 1891)	2013 (1021, 4199)	< 0.001	< 0.001
Urine ACR (mg/g)	277 (53, 1636)	118 (47, 510)	61 (12, 290)	134 (31, 677)	405 (79, 1755)	1982 (630, 4150)	< 0.001	< 0.001

*ACEIs* angiotensin-converting-enzyme in inhibitors, *ACR* albumin/creatinine ratio, *ARBs* angiotensin receptor blockers, *BMI* body mass index, *CCB* calcium channel blocker, *CKD* chronic kidney disease, *eGFR* estimated glomerular filtration rate, *GBTM* group-based trajectory modelling, *IQR* inter-quartile range, *NSAID* nonsteroidal anti-inflammatory drugs* OAD* oral antidiabetic, *PCR* protein/creatinine ratio, *T-CHO* total cholesterol, *TG* triglyceride.

^†^*p *values are calculated by Kruskal–Wallis test for continuous variables and Chi-square test for categorical variables.

^‡^*p *values for trend are calculated by Spearman's correlation for continuous variables and by Cochran-Armitage trend test for binary variables.

### Clinical characteristics by the responsiveness to pre-ESRD program

**Figure 2:**
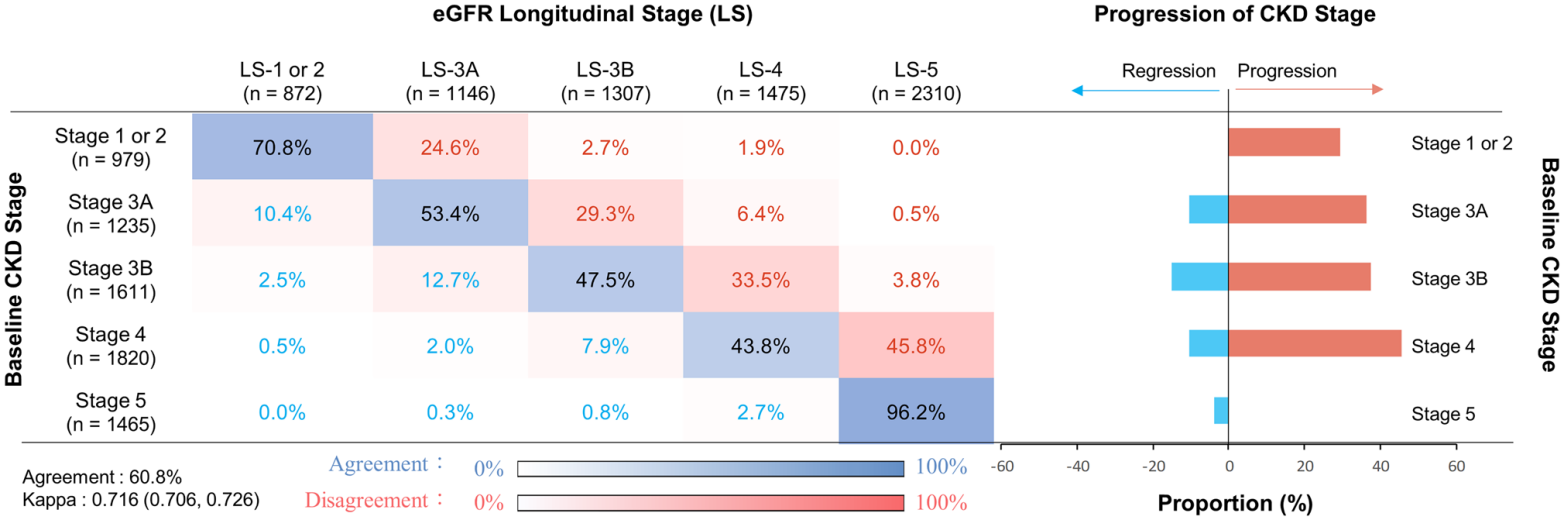
Concordance of baseline CKD stage and longitudinal stages of eGFR trajectory.

**Figure 3:**
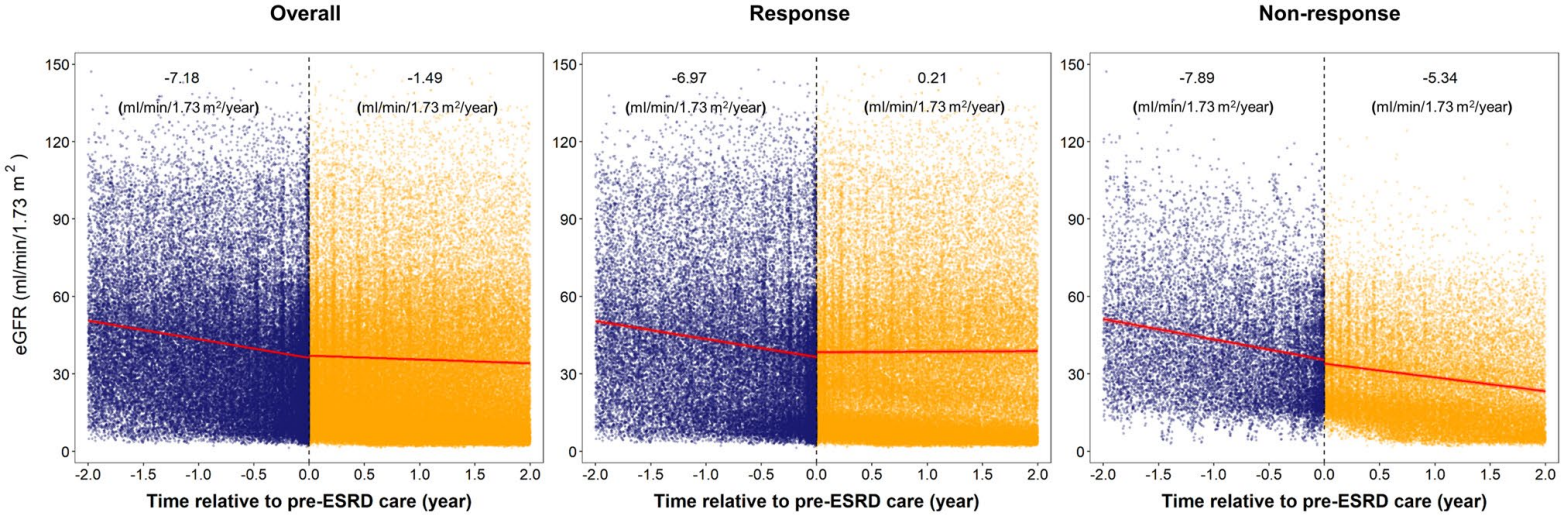
The eGFR slope (red line), with the light red shaded area representing the 95% confidence interval, before and after enrollment of the entire study population into the pre-ESRD program. The eGFR slope was modeled using the growth piecewise linear mixed model through the incorporation of random effects. Blue and orange points represent eGFR measurements before and after enrollment into pre-ESRD program, respectively. The estimated value of eGFR slope (mL/min/1.73 m^2^/y) is shown.

Only 3.8% of patients showed improvement in eGFR LS over the follow-up in patients with baseline CKD stage 5 (Fig. 2). Furthermore, the concordance matrix demonstrated a gradual increase in the proportion of CKD stage progression (LS more advanced than baseline CKD stage) with the increase in the baseline eGFR CKD stage (29.2% for stages 1–2; 36.2% for stage 3A; 37.3% for stage 3B; 45.8% for stage 4; Fig. 2). We then divided the study population based on the concordance between baseline CKD stage and eGFR LS into 3 groups: (1) Stage-Stable (4326, 60.8%); (2) Stage-Regression (617, 8.7%); and (3) Stage-Progression (2167, 30.5%). Patients in the Stage-Regression and Stage-Progression groups had comparable baseline eGFR (36.7 vs. 33.5 mL/min/1.73 m^2^, *P* = 0.76; Table 2). However, patients in the Stage-Progression group had significantly higher baseline levels of serum blood urea nitrogen, phosphorus, total cholesterol, and uPCR along with significantly lower baseline levels of serum albumin, hemoglobin, and urine creatinine compared with those in the other groups (Table 2). Also, responsive patients were more likely to have been exposed to NSAIDs and have better baseline levels of S-Cre, blood urea nitrogen, SUA, phosphorus, and uPCR than nonresponsive patients (Table S2). The eGFR slopes before enrollment to pre-dialysis care were comparable between responsive and nonresponsive patients (− 6.97 vs. − 7.89 mL/min/1.73 m^2^/y). The eGFR slope was then flattened to 0.21 mL/min/1.73 m^2^/y among responsive patients, whereas the annual eGFR slope kept declining at − 5.34 mL/min/1.73 m^2^/y in nonresponsive patients (Fig. 3). The dramatic slowness of declining eGFR slope was consistently observed for patients with baseline CKD stage 3b-5 after enrollment to pre-ESRD program (Figure S2).

**Table 2:** Baseline demographic and clinical characteristics according to the concordance of baseline CKD stage and eGFR LS.

	Group A: Agreement	Group B: Improving	Group C: Deteriorating	Group A versus B *P* value	Group A versus C *P *value	Group B versus C *P *value
**N (%)**	**4326 (60.8)**	**617 (8.7)**	**2167 (30.5)**			
**Demographic**						
Age, median (IQR)	67.5 (57.2, 76.3)	68.5 (57.7, 77.3)	67.6 (56.9, 76.2)	0.119	0.914	0.157
BMI, median (IQR)	24.2 (22.0, 26.9)	24.6 (22.2, 27.3)	24.6 (22.2, 27.3)	0.151	0.004	0.782
Female, n (%)	1926 (44.5)	206 (33.4)	893 (41.2)	< 0.001	0.011	0.001
CKD stage, n (%)						
1	275 (6.4)	1 (0.2)	18 (0.8)	< 0.001	< 0.001	< 0.001
2	349 (8.1)	8 (1.3)	173 (8.0)			
3	1594 (36.9)	400 (64.8)	1126 (52.0)			
4	853 (19.8)	169 (27.4)	815 (37.7)			
5	1246 (28.9)	39 (6.3)	32 (1.5)			
Diabetes, n (%)	1466 (33.9)	188 (30.5)	967 (44.6)	0.090	< 0.001	< 0.001
Hypertension, n (%)	2732 (63.2)	363 (58.8)	1512 (69.8)	0.036	< 0.001	< 0.001
Cardiovascular disease, n (%)	1549 (35.8)	244 (39.6)	881 (40.7)	0.073	0.000	0.615
**Medication, n (%)**						
Pentoxifylline	1030 (24.4)	131 (21.6)	587 (27.6)	0.121	0.007	0.003
NSAIDs	1062 (25.2)	209 (34.4)	554 (26.0)	< 0.001	0.473	< 0.001
Contrast	371 (8.8)	70 (11.5)	207 (9.7)	0.030	0.228	0.196
*Anti-platelet*	1412 (33.5)	199 (32.7)	805 (37.8)	0.716	0.001	0.022
Aspirin	1134 (26.9)	169 (27.8)	651 (30.6)	0.636	0.002	0.189
Dipyridamole	280 (6.6)	29 (4.8)	148 (7.0)	0.079	0.642	0.054
Other Anti-platelet agents	1231 (29.2)	185 (30.4)	708 (33.2)	0.529	0.001	0.192
*Urate-lowering agents*	1086 (25.8)	189 (31.1)	559 (26.2)	0.005	0.669	0.018
Allopurinol	477 (11.3)	73 (12.0)	249 (11.7)	0.613	0.652	0.831
Febuxostat	93 (2.2)	19 (3.1)	40 (1.9)	0.159	0.391	0.062
Benzbromarone	448 (10.6)	87 (14.3)	252 (11.8)	0.007	0.146	0.102
Colchicine	486 (11.5)	87 (14.3)	251 (11.8)	0.047	0.758	0.095
Sulfinpyrazone	41 (1.0)	12 (2.0)	24 (1.1)	0.027	0.563	0.106
*Anti-hypertension agents*	3368 (79.9)	478 (78.6)	1830 (85.9)	0.481	< 0.001	< 0.001
ACEIs	847 (20.1)	94 (15.5)	509 (23.9)	0.007	0.001	< 0.001
ARBs	1786 (42.3)	221 (36.4)	1061 (49.8)	0.005	< 0.001	< 0.001
Trichlorethiazide	396 (9.4)	60 (9.9)	232 (10.9)	0.705	0.058	0.471
Furosemide, Spironolactone, Amizide, Indapamide	1705 (40.4)	260 (42.8)	988 (46.4)	0.272	< 0.001	0.114
α blocker	864 (20.5)	109 (17.9)	502 (23.6)	0.142	0.005	0.003
β blocker	1484 (35.2)	222 (36.5)	883 (41.5)	0.521	< 0.001	0.029
CCB	1985 (47.1)	247 (40.6)	1133 (53.2)	0.003	< 0.001	< 0.001
*Anti-diaetes agents*	1542 (36.6)	193 (31.7)	991 (46.5)	0.021	< 0.001	< 0.001
Oral hypoglycemic agents (OAD)	1235 (29.3)	157 (25.8)	795 (37.3)	0.079	< 0.001	< 0.001
Insuline	727 (17.2)	95 (15.6)	493 (23.2)	0.323	< 0.001	< 0.001
**Baseline biochemical parameters, median (IQR)**						
eGFR (mL/min/1.73 m^2^)	29.3 (12.3, 52.3)	36.7 (25.6, 44.6)	33.5 (20.8, 48.6)	< 0.001	< 0.001	0.760
Serum creatinine (mg/dL)	1.94 (1.33, 4.03)	1.77 (1.48, 2.30)	1.82 (1.37, 2.60)	0.000	< 0.001	0.774
Blood urea nitrogen (mg/dL)	31.0 (19.0, 53.0)	25.0 (19.0, 36.0)	28.0 (20.0, 39.0)	< 0.001	< 0.001	0.001
Serum uric acid (mg/dL)	7.30 (6.00, 8.60)	7.20 (6.05, 8.80)	7.30 (6.20, 8.50)	0.587	0.645	0.838
Sodium (mmol/L)	138 (136, 140)	138 (135, 140)	138 (136, 140)	0.109	0.927	0.137
Potassium (mmol/L)	4.20 (3.80, 4.60)	4.15 (3.70, 4.60)	4.20 (3.90, 4.60)	0.000	0.436	0.002
Calcium (mg/dL))	8.90 (8.40, 9.20)	9.10 (8.60, 9.40)	8.90 (8.50, 9.20)	< 0.001	0.985	< 0.001
Phosphate (mg/dL)	4.10 (3.50, 4.80)	3.80 (3.20, 4.30)	4.00 (3.50, 4.40)	< 0.001	< 0.001	< 0.001
Serum Albumin (g/dL)	3.90 (3.50, 4.30)	4.00 (3.60, 4.40)	3.80 (3.40, 4.20)	0.001	< 0.001	< 0.001
Hemoglobin (g/dL)	11.1 (9.4, 13.0)	12.1 (10.3, 13.6)	11.2 (9.8, 12.8)	< 0.001	0.020	< 0.001
T-CHO (mg/dL)	179 (154, 210)	180 (152, 213)	185 (156, 216)	0.795	0.000	0.074
TG (mg/dL)	128 (89, 188)	134 (91, 202)	134 (92, 201)	0.093	0.000	0.639
Urine creatinine (mg/dL)	84 (55, 127)	99 (60, 148)	82 (54, 119)	< 0.001	0.015	< 0.001
Urine PCR (mg/g)	713 (199, 1900)	183 (89, 545)	1161 (323, 3472)	< 0.001	< 0.001	< 0.001
Urine ACR (mg/g)	205 (47, 1094)	49 (16, 206)	860 (138, 3015)	< 0.001	< 0.001	< 0.001

*ACEIs* angiotensin-converting-enzyme in inhibitors, *ACR* albumin/creatinine ratio, *ARBs* angiotensin receptor blockers, *BMI* body mass index, *CCB1* calcium channel blocker, *CKD* chronic kidney disease, *eGFR* estimated glomerular filtration rate, *GBTM1* group-based trajectory modelling, *IQR* inter-quartile range, *NSAID* nonsteroidal anti-inflammatory drugs, *OAD* oral antidiabetic, *PCR* protein/creatinine ratio, *T-CHO* total cholesterol, *TG* triglyceride.

*P *values are calculated by Kruskal–Wallis test for continuous variables and Chi-square test for categorical variables.

### Clinical prognosis of eGFR LS and the responsiveness to pre-ESRD program

Over the 21,662.36 and 33,472.81 person-years of follow-up, 1451 ESRD events and 2031 deaths occurred, respectively. ESRD and all-cause mortality incidence were 66.9 and 60.7 per 1000 person-years, respectively. Compared with patients with eGFR LS-3A, fully adjusted hazard ratios (HRs) for dialysis and all-cause mortality among patients with eGFR LS-5 were 70.0 (95% confidence interval [CI] 27.8–176.7) and 3.69 (95% CI 2.71–5.04), respectively. On the basis of parametric survival models, the adjusted times to the outcomes of ESRD and all-cause mortality in patients with eGFR LS-5 were 92% (95% CI 86–96%) and 57% (95% CI 48–65%) shorter, respectively, than those with eGFR LS-3A (Table 3). The adjusted HRs for ESRD and all-cause mortality of nonresponsive patients were 2.17 (95% CI 1.9–2.5) and 1.42 (95% CI 1.27–1.59), respectively, compared with those who responded to pre-dialysis care. From the parametric perspective, the adjusted time to ESRD and all-cause death in nonresponsive patients was 39% (95% CI 33–44%) and 20% (95% CI 14–26%) shorter, respectively, than that in responsive patients (Table 3). In the subgroup analysis, we found men and patients with an uPCR < 500 mg/g creatinine or a baseline serum phosphorus < 4 mg/dL who showed no response to pre-dialysis care were more vulnerable to develop ESRD (Table S3).

**Table 3:** HRs (95% CIs) and event time ratio (ETRs, 95% CIs) of the risk of progression to ESRD and all-cause mortality based on trajectories and responsiveness (parametric survival modeling under Weibull regression).

	Crude model			Adjusted model		
	N	HR (95% CI)	ETR (95% CI)	N	HR (95% CI)	ETR (95% CI)
*Dialysis*						
**Baseline eGFR**						
CKD stage 1 or 2 (eGFR ≥ 60)	979	0.78 (0.46, 1.35)	1.181 (0.815, 1.712)	773	0.48 (0.26, 0.89)	1.599 (1.079, 2.371)
CKD stage 3A (45 ≤ eGFR < 60)	1235	1.00 (Ref)	Ref	942	1.00 (Ref)	Ref
CKD stage 3B (30 ≤ eGFR < 45)	1611	2.62 (1.77, 3.87)	0.518 (0.396, 0.677)	1198	2.39 (1.53, 3.75)	0.569 (0.426, 0.761)
CKD stage 4 (15 ≤ eGFR < 30)	1820	8.82 (6.16, 12.64)	0.226 (0.175, 0.291)	1425	8.04 (5.34, 12.11)	0.261 (0.199, 0.342)
CKD stage 5 (eGFR < 15)	1465	42.36 (29.75, 60.31)	0.077 (0.060, 0.100)	1092	38.84 (25.92, 58.21)	0.094 (0.072, 0.124)
**eGFR trajectory**						
CKD stage 1 or 2	876	0.19 (0.02, 1.58)	2.917 (0.741, 11.487)	683	0.34 (0.04, 2.91)	1.927 (0.525, 7.071)
CKD stage 3A	1153	1.00 (Ref)	Ref	835	1.00 (Ref)	Ref
CKD stage 3B	1308	1.98 (0.82, 4.77)	0.640 (0.360, 1.138)	991	1.55 (0.54, 4.43)	0.767 (0.408, 1.442)
CKD stage 4	1477	18.96 (8.91, 40.39)	0.146 (0.089, 0.241)	1154	9.75 (3.91, 24.30)	0.254 (0.146, 0.442)
CKD stage 5	2321	164.88 (78.42, 346.66)	0.036 (0.022, 0.058)	1767	70.03 (27.75, 176.70)	0.077 (0.044, 0.137)
**Disease progression status**						
Response	4943	1.00 (Ref)	Ref	3695	1.00 (Ref)	Ref
Non-response	2167	1.30 (1.16, 1.44)	0.815 (0.748, 0.887)	1735	2.17 (1.89, 2.49)	0.611 (0.558, 0.668)
*All-cause mortality*						
**Baseline eGFR**						
CKD stage 1 or 2 (eGFR ≥ 60)	979	0.63 (0.48, 0.81)	1.417 (1.167, 1.722)	773	0.96 (0.71, 1.30)	1.027 (0.843, 1.250)
CKD stage 3A (45 ≤ eGFR < 60)	1235	1.00 (Ref)	Ref	942	1.00 (Ref)	Ref
CKD stage 3B (30 ≤ eGFR < 45)	1611	1.69 (1.41, 2.04)	0.675 (0.588, 0.774)	1198	1.38 (1.11, 1.71)	0.810 (0.702, 0.935)
CKD stage 4 (15 ≤ eGFR < 30)	1820	2.88 (2.43, 3.41)	0.454 (0.399, 0.517)	1425	2.26 (1.85, 2.75)	0.585 (0.513, 0.668)
CKD stage 5 (eGFR < 15)	1465	3.16 (2.66, 3.74)	0.424 (0.372, 0.484)	1092	2.95 (2.41, 3.61)	0.491 (0.428, 0.564)
**eGFR trajectory**						
CKD stage 1 or 2	876	0.56 (0.41, 0.77)	1.534 (1.209, 1.947)	683	0.77 (0.52, 1.14)	1.184 (0.917, 1.531)
CKD stage 3A	1153	1.00 (Ref)	Ref	835	1.00 (Ref)	Ref
CKD stage 3B	1308	2.05 (1.66, 2.52)	0.587 (0.503, 0.686)	991	1.39 (1.07, 1.80)	0.806 (0.680, 0.955)
CKD stage 4	1477	3.19 (2.63, 3.87)	0.421 (0.364, 0.488)	1154	2.20 (1.68, 2.87)	0.597 (0.500, 0.712)
CKD stage 5	2321	4.02 (3.35, 4.83)	0.355 (0.308, 0.409)	1767	3.69 (2.71, 5.04)	0.425 (0.346, 0.522)
**Disease progression status**						
Response	4943	1.00 (Ref)	Ref	3695	1.00 (Ref)	Ref
Non-response	2167	1.28 (1.17, 1.41)	0.834 (0.780, 0.891)	1735	1.42 (1.27, 1.59)	0.795 (0.738, 0.857)

Adjusted model: adjusted for age at entry, gender, BMI, smoking status, alcohol consumption, education, diabetes, hypertension, cardiovascular disease, baseline medication for NSAIDs, anti-platelet agents, urate-lowering agents, ACEIs/ARBs, Diuretics, baseline biochemical parameters for pooled urine PCR, and eGFR.

*ACEI* angiotensin-converting-enzyme in inhibitors, *ARBs* angiotensin receptor blockers, *BMI* body mass index, *CI* confidence interval, *CKD* chronic kidney disease, *eGFR* estimated glomerular filtration rate, *ETR* event time ratio, *ESRD* end stage renal disease, *HR* hazard ratio, *IQR* inter-quartile range, *NSAID* nonsteroidal anti-inflammatory drugs, *PCR* protein/creatinine ratio.

### Prediction of responsiveness to the pre-ESRD program

**Figure 4:**
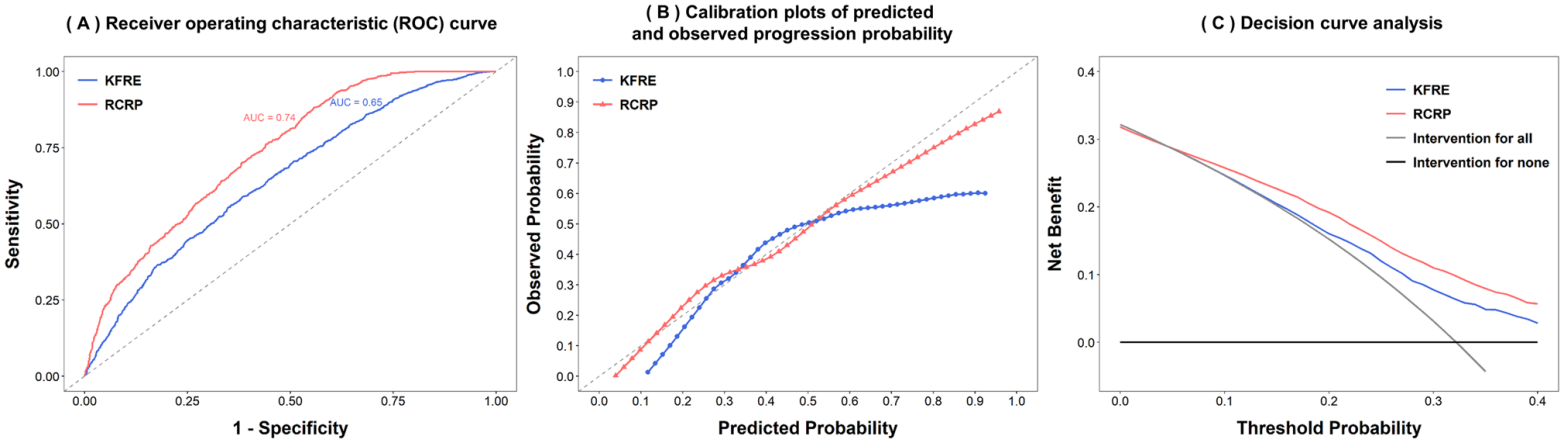
Prediction performance of the proposed renal care responsiveness prediction (RCRP) model compared with the reference model using seven variables based on kidney failure risk estimation (KFRE). (**A**) Receiver operating characteristic curve, (**B**) calibration plots of predicted and observed progression probability, and (**C**) decision curve analysis.

The significant positive associations of male sex, diabetes, hypertension, and uPCR level with nonresponsiveness to pre-dialysis care were identified in the multiple logistic regression. By contrast, baseline eGFR, age, NSAID use, hemoglobin, and serum albumin were negatively associated with nonresponsiveness to pre-dialysis care (Table 4). Compared with the conventional Kidney Failure Risk Equation (KFRE) 7-variable model, our proposed prediction model for responsiveness to pre-dialysis care using baseline demographics, comorbidities, medications, and biochemical profiles performed significantly better in terms of discrimination and calibration, with an area under the receiver operating characteristics of 0.74 (Fig. 4A, B). In decision curve analysis, our proposed model correctly identified an additional 3 nonresponsiveness patients for every 100 patients with CKD at the threshold probability of 20% compared with the KFRE 7-variable model (Fig. 4C).

**Table 4:** Factors associated with nonresponsiveness to pre-ESRD care based on multiple logistic regression in the crude model, full model (with and without imputation), KFRE (Kidney Failure Risk Estimation), and RCRP (Renal Care Responsiveness Prediction).

	N	Crude model	Full model (N = 3001)	Full model (MI) (N = 7110)	KFRE (N = 4080)	RCRP (N = 3360)
		Odds ratio (95% CI)	Odds ratio (95% CI)	Odds ratio (95% CI)	Odds ratio (95% CI)	Odds ratio (95% CI)
**Demographic**						
Age	7110	1.000 (0.996, 1.003)	1.001 (0.993, 1.008)	0.997 (0.992, 1.003)	0.999 (0.994, 1.005)	0.994 (0.988, 1.000)
Male	7110	1.082 (0.977, 1.199)	1.178 (0.966, 1.437)	1.100 (0.963, 1.256)	1.027 (0.893, 1.183)	**1.649 (1.382, 1.972)**
BMI	6951	1.017 (1.005, 1.029)	**1.027 (1.006, 1.048)**	1.006 (0.993, 1.021)		
Smoking, former (Ref: Never)	7110	0.951 (0.781, 1.151)	**0.601 (0.382, 0.932)**	0.771 (0.583, 1.020)		
Smoking, current (Ref: Never)		1.036 (0.872, 1.227)	0.908 (0.677, 1.212)	0.975 (0.803, 1.184)		
Alcohol consumption, former (Ref: Never)	7110	1.116 (0.889, 1.394)	1.513 (0.940, 2.446)	1.276 (0.927, 1.757)		
Alcohol consumption, current (Ref: Never)		0.931 (0.700, 1.225)	0.766 (0.449, 1.273)	0.905 (0.668, 1.227)		
Education level (year), 9 ≤ ~ < 12 (Ref: < 9)	7110	1.024 (0.900, 1.165)	1.012 (0.819, 1.252)	1.020 (0.887, 1.173)		
Education level (year), 12 ≤ ~ < 16 (Ref: < 9)		1.076 (0.931, 1.242)	1.102 (0.848, 1.434)	1.101 (0.929, 1.303)		
Education level (year), 16 + (Ref: < 9)		1.009 (0.845, 1.204)	1.054 (0.757, 1.463)	1.069 (0.871, 1.312)		
Diabetes	7106	1.602 (1.445, 1.777)	**1.419 (1.183, 1.701)**	**1.445 (1.258, 1.661)**		**1.314 (1.109, 1.557)**
Hypertension	7106	1.378 (1.237, 1.536)	0.981 (0.792, 1.216)	1.086 (0.945, 1.248)		**1.213 (1.011, 1.457)**
Cardiovascular disease	7106	1.203 (1.085, 1.334)	1.029 (0.840, 1.259)	1.061 (0.924, 1.219)		
**Medication**						
NSAIDs	6956	0.983 (0.875, 1.104)	1.154 (0.962, 1.383)	0.918 (0.809, 1.043)		1.016 (0.851, 1.212)
Anti-platelet	6956	1.212 (1.090, 1.348)	0.961 (0.784, 1.178)	1.003 (0.876, 1.147)		
Urate-lowering agents	6956	0.991 (0.882, 1.112)	0.929 (0.768, 1.123)	1.059 (0.930, 1.206)		
ACEIs/ARBs	6956	1.480 (1.333, 1.643)	**1.329 (1.092, 1.619)**	**1.212 (1.061, 1.384)**		
Trichlorethiazide	6956	1.263 (1.140, 1.399)	0.959 (0.792, 1.160)	1.028 (0.904, 1.169)		
Pentoxifylline	6956	1.201 (1.069, 1.348)	1.072 (0.897, 1.279)	1.103 (0.968, 1.257)		
**Baseline biochemical parameters**						
eGFR (mL/min/1.73 m^2^)	7110	1.001 (0.998, 1.003)	1.000 (0.994, 1.006)	**0.988 (0.984, 0.992)**	**1.010 (1.006, 1.014)**	
Pooled urine PCR (per 100 mg/g cre)	5669	1.013 (1.011, 1.015)	**1.013 (1.009, 1.016)**	1.007 (0.995, 1.018)	**1.014 (1.011, 1.017)**	**1.016 (1.013, 1.020)**
Calcium (mg/dL))	5319	0.977 (0.892, 1.069)	1.020 (0.876, 1.188)	1.052 (0.930, 1.190)	**1.135 (1.001, 1.287)**	0.962 (0.826, 1.120)
Phosphate (mg/dL)	4935	0.808 (0.756, 0.862)	**0.861 (0.773, 0.959)**	0.919 (0.843, 1.001)	**0.766 (0.704, 0.833)**	0.966 (0.870, 1.070)
Serum Albumin (g/dL)	5857	0.713 (0.654, 0.776)	0.901 (0.761, 1.068)	**0.793 (0.649, 0.970)**	0.885 (0.772, 1.015)	1.074 (0.913, 1.264)
Hemoglobin (g/dL)	5477	1.011 (0.986, 1.036)	**0.937 (0.892, 0.983)**	**0.944 (0.910, 0.980)**		**0.852 (0.814, 0.892)**
Serum creatinine (mg/dL)	7110	0.744 (0.715, 0.772)				**0.485 (0.445, 0.527)**
Blood urea nitrogen (mg/dL)	6613	0.986 (0.983, 0.988)	**0.971 (0.965, 0.977)**	**0.969 (0.965, 0.974)**		
Serum uric acid (mg/dL)	6294	0.994 (0.968, 1.021)	1.025 (0.982, 1.070)	1.027 (0.997, 1.059)		
T-CHO (mg/dL)	6018	1.001 (1.000, 1.002)	0.999 (0.998, 1.001)	1.000 (0.999, 1.002)		

*ACEIs* Angiotensin-converting-enzyme inhibitors, *ARBs* Angiotensin II receptor blockers, *BMI* body mass index, CI confidence interval, CKD chronic kidney disease, eGFR estimated glomerular filtration rate, ETR event time ratio, ESRD end stage renal disease, NSAID nonsteroidal anti-inflammatory drugs, PCR protein/creatinine ratio, RCRP renal care responsiveness prediction.

Odds ratios labeled in bold are statistically significant at the level of alpha = 0.05.

## Discussion

eGFR LSs were largely determined based on the baseline CKD stage when the patients were enrolled into the pre-ESRD program. The more advanced the baseline CKD stage, the less likely the patient was to remain in the baseline stage longitudinally even under the nephrologists’ care. The responsiveness to the nephrologists’ care based on the discrepancy between baseline CKD stage and eGFR-LS was well validated according to the improvement of eGFR declining slope before and after the pre-dialysis enrollment. Patients with poor responsiveness have significantly shorter dialysis-free survival time and higher mortality compared with those whose progression was halted by the pre-ESRD program. Our proposed complete predictive model outperformed the conventional KFRE in predicting CKD progression even under pre-dialysis care.

The fast-decline characteristic of eGFR trajectory was not observed among patients with advanced CKD under nephrologists’ care. Along with the slow declining eGFR slope after the pre-ESRD enrollment, the multidisciplinary pre-ESRD care program of Taiwan did halt the CKD progression; however, patients with CKD stage 5 at baseline still experienced rapid progression to ESRD with a much shorter median dialysis-free survival time of, for instance, approximately 5 months if the median dialysis-free survival time for patients with LS-3A eGFR is 5 years. The dialysis-free survival time were comparable with those of the Initiating Dialysis Early and Late randomized controlled trial^28^. Such rapid and irreversible progression to ESRD makes shared-decision making regarding the issues of dialysis modality and access preparation essential in pre-dialysis care for patients with stage 5 CKD at baseline. Despite the kidney function of patients with CKD under appropriate pre-dialysis care had been generally stabilized against rapid progression, the steep declining eGFR slopes before pre-dialysis care were observed particularly among patients with stages 4 and 5 CKD at baseline indicating rapid loss of kidney function before the enrollment of pre-ESRD program (Figure S2). This observation highlighted the importance of determining a method to detect and refer patients with early-stage CKD to the pre-ESRD program using innovative diagnostic approaches, such as a noninvasive CKD screening through portable ultrasound^29^.

Some studies have identified potential predictors of rapid CKD progression and CKD regression among diverse populations. In a study of 949 African American patients, only 3.3% of patients had a clearly improving eGFR slope over a 12-year follow-up. Lower proteinuria and blood pressure were associated with improved eGFR^30^. Another study conducted in France showed that 15.3% of 394 patients had improved eGFR measurements within a 2-year follow-up period even among patients with stage 4 or 5 CKD at baseline. Patients who showed improvement had lower urinary albumin-to-creatinine ratio (uACR) than those who did not^31^. Consistently, Borrelli et al. reported that 25% of patients with an eGFR range of 15–60 mL/min/1.73 m^2^ at baseline exhibited CKD regression under nephrologists’ care. Factors associated with CKD regression were low proteinuria, low blood pressure, high BMI, and absence of autosomal polycystic kidney disease^32^. A large study of 36,195 patients with stage 3 CKD in the United States showed that the key driving factors of accelerated CKD progression defined as a loss of eGFR > 4 mL/min/1.73 m^2^/y include proteinuria, high blood pressure, heart failure, anemia, and older age, regardless of diabetic status^33^. Another large study conducted in Hong Kong concluded that microalbuminuria and retinopathy are associated with an accelerated decline in eGFR defined as joint-latent class modeling among patients with diabetes with baseline eGFR > 60 mL/min/1.73 m^2^^15^. However, the method of integrating these findings into daily practice is uncertain. In our study, we found that although the KFRE is useful and robust in predicting the risk of progression to ESRD, it is of little value for distinguishing nonresponders from responders to the nephrologists’ care^34^. Although eGFR variability based on serial eGFR measurements helps predict the responsiveness of patients to pre-ESRD programs, this predictor requires an additional observation period of, for instance, 1 year after the enrollment of pre-ESRD program to obtain multiple eGFR measurements^35^. To practically help clinicians predict nonresponders in a single clinical encounter, we proposed a predictive model, albeit complex, using variables that are required at the enrollment to pre-dialysis care in Taiwan and readily available in EMR to provide real-time prediction of care responsiveness. The moderate performance of both KFER and ours implied the difficulty in predicting clinical response to nephrologists’ care in the present population. We found men, patients without significant proteinuria, and those with relatively good baseline phosphorus control, who showed no response to pre-dialysis care pose a critical challenge to nephrologists as they are particularly vulnerable to rapid progression to ESRD. More targeted research efforts are needed to identify effective therapeutic strategies for in-time diagnosis and treatment of nonresponders.

The present study has several limitations. First, we did not validate the predictive performance of our proposed model in other populations, which would also require multiple eGFR measurements to define the CKD progression (status of responsiveness). Second, the study population was derived from the Han-Chinese population and was under a universal healthcare system. Therefore, the predictive model must be generalized to other populations with caution. Third, the possibility of residual confounding, such as medication and dietary nonadherence, and over-adjustment of variables that could be in the causal pathway cannot be completely excluded.

On the basis of discrepancy between baseline CKD stage and LS of eGFR, approximately 60% of the patients with CKD achieved disease stability or improvement in Taiwan’s pre-ESRD care program. Our proposed predictive model improves early identification of responsiveness to pre-dialysis care and facilitates decision sharing between clinicians and patients regarding therapeutic strategies. Large longitudinal databases with multiple eGFR measurements should be used to verify our definitions of disease progression and our model’s prediction validity.

## Supplementary Information

Supplementary Informations.
