# Supplementary material for: Prediction of non-responsiveness to pre-dialysis care program in patients with chronic kidney disease: a retrospective cohort analysis
Source: Sci Rep. 2021 Jul 6;11:13938. doi: 10.1038/s41598-021-93254-0 (PMC8260802; doi:10.1038/s41598-021-93254-0)

**Prediction of Non-Responsiveness to Pre-dialysis Care Program in Patients with Chronic Kidney Disease: A Retrospective Cohort Analysis**

Emily K. King, M.S., Ming-Han Hsieh, M.D., David R. Chang, M.D., Cheng-Ting Lu, M.D., I-Wen Ting, M.D., Charles C.N. Wang, Ph.D., Pei-Shan Chen, M.S., Hung-Chieh Yeh, M.D., Hsiu-Yin Chiang, Ph.D., Chin-Chi Kuo, M.D. Ph.D

**Supplementary Material**

**Table S1.** Missing proportion of variables listed in table 1 (N=7135).

**Table S2.** Baseline Demographic and Clinical Characteristics Based on the Responsiveness of Patients With Stages 3 and 4 CKD at Baseline to Pre-ESRD Care

**Table S3.** Subgroup analysis for associations between non-responsiveness to pre-ESRD program (reference group: responsive) and adverse outcomes according to baseline characteristics. aHR, adjusted hazard ratio; ETR, event time ratio (parametric survival modeling under Weibull regression).

**Figure S1**. The selection process of the study population

**Figure S2.** The eGFR slope (red line), with the light red shaded area representing the 95% confidence interval, before and after enrollment of the study population into the pre-ESRD program stratified by CKD stage. The eGFR slope was modeled using the growth piecewise linear mixed model through the incorporation of random effects. Blue and orange points represent eGFR measurements before and after enrollment into pre-ESRD program, respectively. The estimated value of eGFR slope (mL/min/1.73m^2^/y) is shown.

**Supplementary Methods**

***Co-variables***

Sociodemographic variables collected during the enrollment interview included age, race/ethnicity, sex, education, cigarette smoking, and alcohol consumption. Smoking and alcohol consumption were categorized as current, former, and never. Body mass index (BMI) was calculated as weight in kilograms divided by height in meters squared. Diabetes mellitus and hypertension were defined based on physicians’ clinical diagnoses according to International Classification of Diseases, ninth revision (*ICD-9*) codes or the use of blood-pressure- and glucose-lowering agents. A history of cardiovascular disease (CVD) was defined as documented coronary artery disease, myocardial infarction, stroke, or heart failure based on *ICD-9* codes in EMRs 1 year before enrollment to the pre-ESRD program.

**Table S1.** Missing proportion of variables listed in table 1 (N=7135).

| **Variables** | **Missing N (%)** |
| --- | --- |
| Body mass index (kg/m^2^) | 164 (2.3%) |
| Initial CKD stage | 12 (0.2%) |
| Diabetes | 10 (0.1%) |
| Hypertension | 10 (0.1%) |
| Cardiovascular disease | 10 (0.1%) |
| **Baseline medication profiles** | 163 (2.3%) |
| Pentoxifylline | 163 (2.3%) |
| NSAIDs | 163 (2.3%) |
| Contrast media | 163 (2.3%) |
| *Anti-platelet* | 163 (2.3%) |
| *Urate-lowering agents* | 163 (2.3%) |
| *Anti-hypertension agents* | 163 (2.3%) |
| *Anti-diaetes agents* | 163 (2.3%) |
| **Baseline biochemical profiles** | 163 (2.3%) |
| Blood urea nitrogen (mg/dL) | 522 (7.3%) |
| Serum uric acid (mg/dL) | 839 (11.8%) |
| Sodium (mmol/L) | 1224 (17.2%) |
| Potassium (mmol/L) | 691 (9.7%) |
| Calcium (mg/dL)) | 1816 (25.5%) |
| Phosphate (mg/dL) | 2200 (30.8%) |
| Serum Albumin (g/dL) | 1278 (17.9%) |
| Hemoglobin (g/dL) | 1657 (23.2%) |
| Total cholesterol (mg/dL) | 1115 (15.6%) |
| Triglyceride (mg/dL) | 863 (12.1%) |
| Urine creatinine (mg/dL) | 1270 (17.8%) |
| Urine PCR (mg/g) | 2001 (28.0%) |
| Urine ACR (mg/g) | 5002 (70.1%) |

**Abbreviations:** ACR, albumin/creatinine ratio; CKD, chronic kidney disease; NSAID, nonsteroidal anti-inflammatory drugs; PCR, protein/creatinine ratio.

**Table S2.** Baseline Demographic and Clinical Characteristics Based on the Responsiveness of Patients With Stages 3 and 4 CKD at Baseline to Pre-ESRD Care

|  | **Agreement / Improving (n= 3016, 60.8%)** | **Disagreement-deteriorating (n= 1941, 39.2%)** | **P-value** |
| --- | --- | --- | --- |
| **Demographic** |  |  |  |
| Age, median (IQR) | 70.4 (60.4, 77.8) | 68.0 (57.1, 76.4) | <.0001 |
| BMI, median (IQR) | 24.4 (22.2, 27.0) | 24.5 (22.2, 27.2) | 0.396 |
| Gender, n (%) |  |  |  |
| Female | 1069 (35.4) | 804 (41.4) | <.0001 |
| Male | 1947 (64.6) | 1137 (58.6) |  |
| CKD stage, n (%) |  |  |  |
| 3 | 1994 (66.1) | 1126 (58.0) | <.0001 |
| 4 | 1022 (33.9) | 815 (42.0) |  |
| Diabetes, n (%) | 929 (30.8) | 853 (44.0) | <.0001 |
| Hypertension, n (%) | 1893 (62.8) | 1351 (69.6) | <.0001 |
| Cardiovascular disease, n (%) | 1228 (40.8) | 812 (41.8) | 0.452 |
| **Medication, n (%)** |  |  |  |
| Pentoxifylline | 747 (25.4) | 540 (28.3) | 0.025 |
| NSAIDs | 872 (29.6) | 503 (26.4) | 0.014 |
| Contrast | 309 (10.5) | 182 (9.5) | 0.278 |
| *Anti-platelet* | 1096 (37.2) | 735 (38.5) | 0.371 |
| Aspirin | 908 (30.8) | 596 (31.2) | 0.781 |
| Dipyridamole | 133 (7.0) | 330 (0.0) | 0.710 |
| other Anti-platelet agents | 987 (33.5) | 650 (34.1) | 0.706 |
| *Urate-lowering agents* | 906 (30.8) | 526 (27.6) | 0.016 |
| Allopurinol | 379 (12.9) | 238 (12.5) | 0.678 |
| Febuxostat | 64 (2.2) | 39 (2.0) | 0.757 |
| Benzbromarone | 412 (14.0) | 238 (12.5) | 0.127 |
| Colchicine | 401 (13.6) | 234 (12.3) | 0.169 |
| Sulfinpyrazone | 39 (1.3) | 22 (1.2) | 0.599 |
| *Anti-hypertension agents* | 2312 (78.5) | 1634 (85.6) | <.0001 |
| ACEI | 543 (18.4) | 442 (23.2) | <.0001 |
| ARBs | 1264 (42.9) | 945 (49.5) | <.0001 |
| Trichlorethiazide | 272 (9.2) | 214 (11.2) | 0.026 |
| Furosemide , Spironolactone, Amizide, Indapamide | 1135 (38.6) | 911 (47.7) | <.0001 |
| α blocker | 593 (20.1) | 457 (23.9) | 0.002 |
| β blocker | 1057 (35.9) | 791 (41.4) | 0.000 |
| CCB | 1280 (43.5) | 1019 (53.4) | <.0001 |
| *Anti-diabetes agents* | 967 (32.9) | 876 (45.9) | <.0001 |
| Oral hypoglycemic agents | 807 (27.4) | 694 (36.4) | <.0001 |
| Insulin | 436 (14.8) | 450 (23.6) | <.0001 |
| **Baseline biochemical parameters, median (IQR)** |  |  |  |
| eGFR (mL/min/1.73m^2^) | 37.0 (25.7, 47.8) | 32.4 (20.5, 46.1) | <.0001 |
| Serum creatinine (mg/dL) | 1.71 (1.41, 2.23) | 1.92 (1.45, 2.66) | <.0001 |
| Blood urea nitrogen (mg/dL) | 25.0 (19.0, 35.0) | 29.0 (21.0, 40.0) | <.0001 |
| Serum uric acid (mg/dL) | 7.30 (6.10, 8.60) | 7.40 (6.30, 8.60) | 0.303 |
| Sodium (mmol/L) | 138 (136, 140) | 138 (136, 140) | 0.041 |
| Potassium (mmol/L) | 4.20 (3.80, 4.60) | 4.20 (3.90, 4.60) | 0.067 |
| Calcium (mg/dL)) | 9.00 (8.70, 9.30) | 8.90 (8.50, 9.20) | <.0001 |
| Phosphate (mg/dL) | 3.80 (3.30, 4.30) | 4.00 (3.50, 4.40) | <.0001 |
| Serum Albumin (g/dL) | 4.00 (3.70, 4.40) | 3.80 (3.40, 4.20) | <.0001 |
| Hemoglobin (g/dL) | 12.0 (10.4, 13.6) | 11.1 (9.8, 12.7) | <.0001 |
| T-CHO (mg/dL) | 179 (154, 209) | 185 (156, 216) | <.0001 |
| TG (mg/dL) | 129 (88, 190) | 134 (91, 201) | 0.002 |
| Urine creatinine (mg/dL) | 96 (63, 143) | 82 (54, 118) | <.0001 |
| Urine PCR (mg/g) | 271 (114, 845) | 1,200 (330, 3,541) | <.0001 |
| Urine ACR (mg/g) | 97 (21, 485) | 988 (147, 3,247) | <.0001 |

***p***-values are calculated by Kruskal-Wallis test for continuous variables and Chi-square test for categorical variables

**Abbreviations:** ACEIs, angiotensin-converting-enzyme in inhibitors; ACR, albumin-to-creatinine ratio; ARBs, angiotensin receptor blockers; BMI, body mass index; CCB, calcium channel blocker; CKD, chronic kidney disease; eGFR, estimated glomerular filtration rate; GBTM, group-based trajectory modelling; IQR, inter-quartile range; NSAID, nonsteroidal anti-inflammatory drugs; PCR, protein-to-creatinine ratio; T-CHO, total cholesterol; TG, triglyceride.

**Table S3.** Subgroup analysis for associations between non-responsiveness to pre-ESRD program (reference group: responsive) and adverse outcomes according to baseline characteristics. aHR, adjusted hazard ratio; ETR, event time ratio (parametric survival modeling under Weibull regression).

|  | **Dialysis** | | **All-cause mortality** | |
| --- | --- | --- | --- | --- |
|  | **aHR  (95% CI)** | **ETR (95% CI)** | **aHR  (95% CI)** | **ETR (95% CI)** |
| **Age** |  |  |  |  |
| <65 years | 1.65 (1.37 - 1.99) | 0.74 (0.67 - 0.83) | 1.39 (1.1 - 1.75) | 0.79 (0.67 - 0.93) |
| ≧65 years | 2.79 (2.22 - 3.5) | 0.52 (0.45 - 0.6) | 1.38 (1.21 - 1.57) | 0.82 (0.75 - 0.89) |
| **Sex** |  |  |  |  |
| Women | **1.66 (1.33 - 2.06)** | **0.75 (0.66 - 0.85)** | 1.53 (1.27 - 1.84) | 0.77 (0.68 - 0.86) |
| Men | **2.54 (2.11 - 3.07)** | **0.55 (0.49 - 0.62)** | 1.31 (1.13 - 1.51) | 0.83 (0.76 - 0.92) |
| **BMI** |  |  |  |  |
| <24 kg/m^2^ | 1.9 (1.55 - 2.34) | 0.66 (0.58 - 0.76) | 1.35 (1.15 - 1.58) | 0.82 (0.73 - 0.91) |
| 24-27 kg/m^2^ | 2.32 (1.72 - 3.13) | 0.62 (0.53 - 0.74) | 1.27 (1.01 - 1.6) | 0.86 (0.75 - 0.99) |
| >27 kg/m^2^ | 1.81 (1.36 - 2.42) | 0.73 (0.63 - 0.85) | 1.48 (1.15 - 1.9) | 0.78 (0.67 - 0.91) |
| **Smoking** |  |  |  |  |
| Never | 2.07 (1.77 - 2.42) | 0.63 (0.57 - 0.7) | 1.48 (1.31 - 1.68) | 0.77 (0.71 - 0.84) |
| Former | 2.6 (1.48 - 4.55) | 0.63 (0.48 - 0.83) | 0.81 (0.49 - 1.33) | 1.14 (0.84 - 1.53) |
| Current | 1.84 (1.2 - 2.83) | 0.74 (0.59 - 0.92) | 1.36 (0.95 - 1.96) | 0.83 (0.66 - 1.04) |
| **Initial CKD stage** |  |  |  |  |
| 3 | 183.93 (10.64 - 3179.09) | 0.16 (0.06 - 0.45) | 2.43 (1.35 - 4.37) | 0.61 (0.43 - 0.86) |
| 4-5 | 2.37 (2.05 - 2.74) | 0.58 (0.53 - 0.64) | 1.43 (1.27 - 1.61) | 0.79 (0.73 - 0.85) |
| **PCR** |  |  |  |  |
| < 500 mg/g creatinine | **7.56 (4.15 - 13.79)** | **0.46 (0.36 - 0.58)** | 1.29 (1.03 - 1.62) | 0.85 (0.74 - 0.98) |
| ≧ 500 mg/g creatinine | **1.72 (1.48 - 2)** | **0.71 (0.64 - 0.78)** | 1.27 (1.11 - 1.46) | 0.85 (0.78 - 0.93) |
| **Diabetes** |  |  |  |  |
| Non-diabetes | **2.11 (1.7 - 2.61)** | **0.65 (0.57 - 0.74)** | 1.39 (1.18 - 1.63) | 0.81 (0.73 - 0.9) |
| Diabetes | **2.04 (1.69 - 2.46)** | **0.64 (0.56 - 0.72)** | 1.4 (1.19 - 1.64) | 0.8 (0.72 - 0.89) |
| **Hypertension** |  |  |  |  |
| Non-hypertension | 1.91 (1.43 - 2.57) | 0.68 (0.57 - 0.81) | 1.32 (1.06 - 1.65) | 0.82 (0.7 - 0.96) |
| Hypertension | 2.29 (1.95 - 2.69) | 0.59 (0.53 - 0.66) | 1.46 (1.28 - 1.66) | 0.79 (0.72 - 0.86) |
| **Cardiovascular disease** |  |  |  |  |
| Non-CVD | 1.97 (1.65 - 2.34) | 0.65 (0.58 - 0.73) | 1.34 (1.14 - 1.57) | 0.82 (0.74 - 0.92) |
| CVD | 2.33 (1.84 - 2.95) | 0.62 (0.54 - 0.71) | 1.47 (1.25 - 1.73) | 0.79 (0.71 - 0.87) |
| **Serum uric acid** |  |  |  |  |
| < 7 mg/dL | 2.18 (1.7 - 2.8) | 0.61 (0.51 - 0.71) | 1.49 (1.24 - 1.79) | 0.78 (0.7 - 0.88) |
| ≧ 7 mg/dL | 2 (1.67 - 2.4) | 0.68 (0.61 - 0.75) | 1.31 (1.12 - 1.53) | 0.83 (0.75 - 0.93) |
| **Serum Phosphorus** |  |  |  |  |
| < 4 mg/dL | **3.44 (2.58 - 4.6)** | **0.51 (0.44 - 0.6)** | 1.48 (1.22 - 1.8) | 0.77 (0.68 - 0.88) |
| ≧ 4 mg/dL | **1.79 (1.47 - 2.18)** | **0.71 (0.64 - 0.8)** | 1.19 (0.99 - 1.43) | 0.89 (0.79 - 1.01) |

**Adjusted model:** adjusted for age at entry, gender, BMI, smoking status, alcohol consumption, education, diabetes, hypertension, cardiovascular disease, baseline medication for NSAIDs, anti-platelet agents, urate-lowering agents, ACEI/ARBs, Diuretics, baseline biochemical parameters for PCR, and eGFR (Sample size: 5430 patients with all co-variables available except for subgroups of serum uric acid and phosphorus, which is 4951 and 3946 in sample size, respectively).

**Abbreviations:** ACEI, angiotensin-converting-enzyme in inhibitors; ARBs, angiotensin receptor blockers; BMI, body mass index; CI, confidence interval; CKD, chronic kidney disease; eGFR, estimated glomerular filtration rate; ETR, event time ratio; ESRD, end stage renal disease; HR, hazard ratio; IQR, inter-quartile range,; NSAID, nonsteroidal anti-inflammatory drugs; PCR, protein-to-creatinine ratio.

**Figure S1**. The selection process of the study population


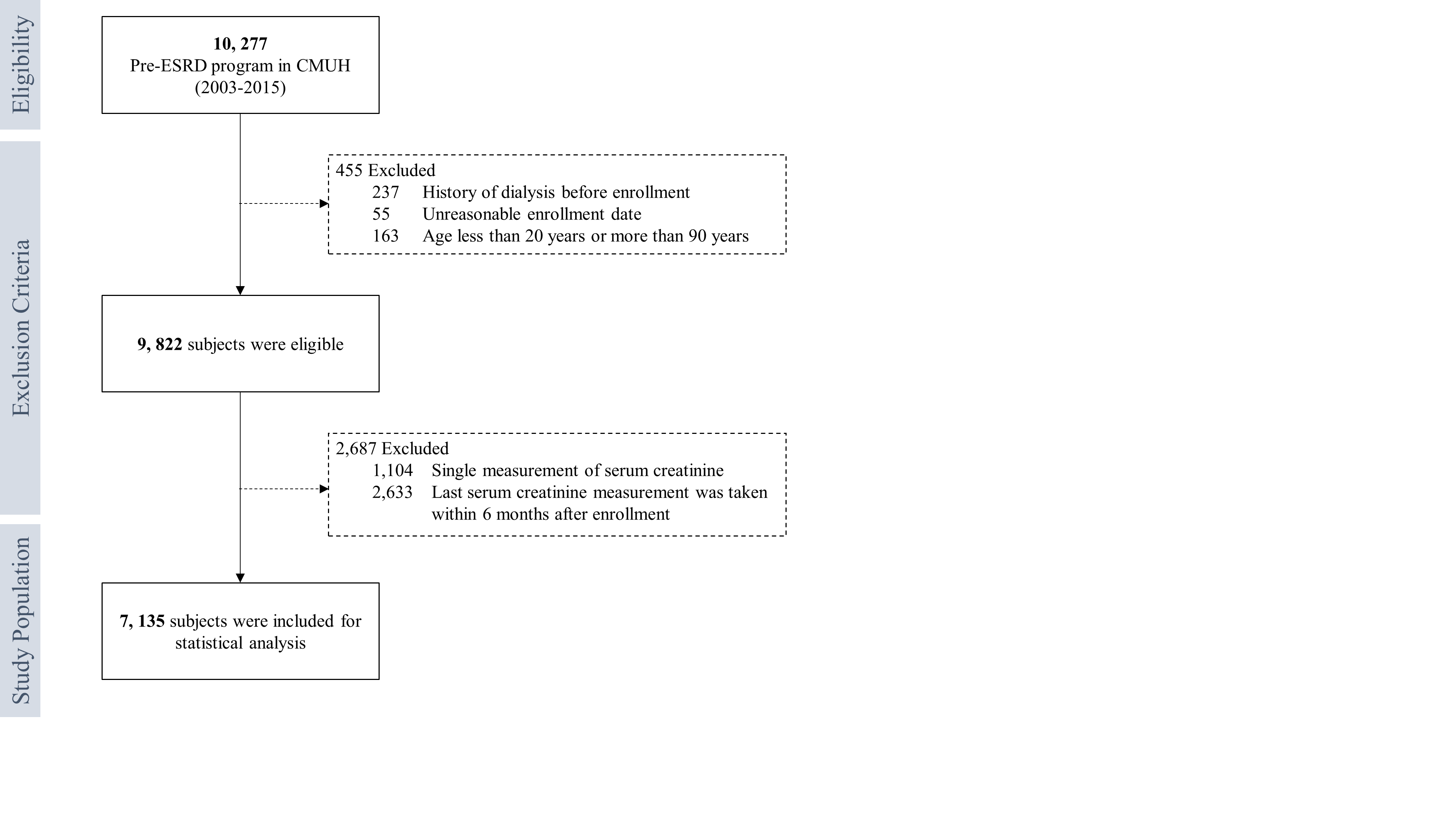


**Figure S2.** The eGFR slope (red line), with the light red shaded area representing the 95% confidence interval, before and after enrollment of the study population into the pre-ESRD program stratified by CKD stage. The eGFR slope was modeled using the growth piecewise linear mixed model through the incorporation of random effects. Blue and orange points represent eGFR measurements before and after enrollment into pre-ESRD program, respectively. The estimated value of eGFR slope (mL/min/1.73m^2^/y) is shown.


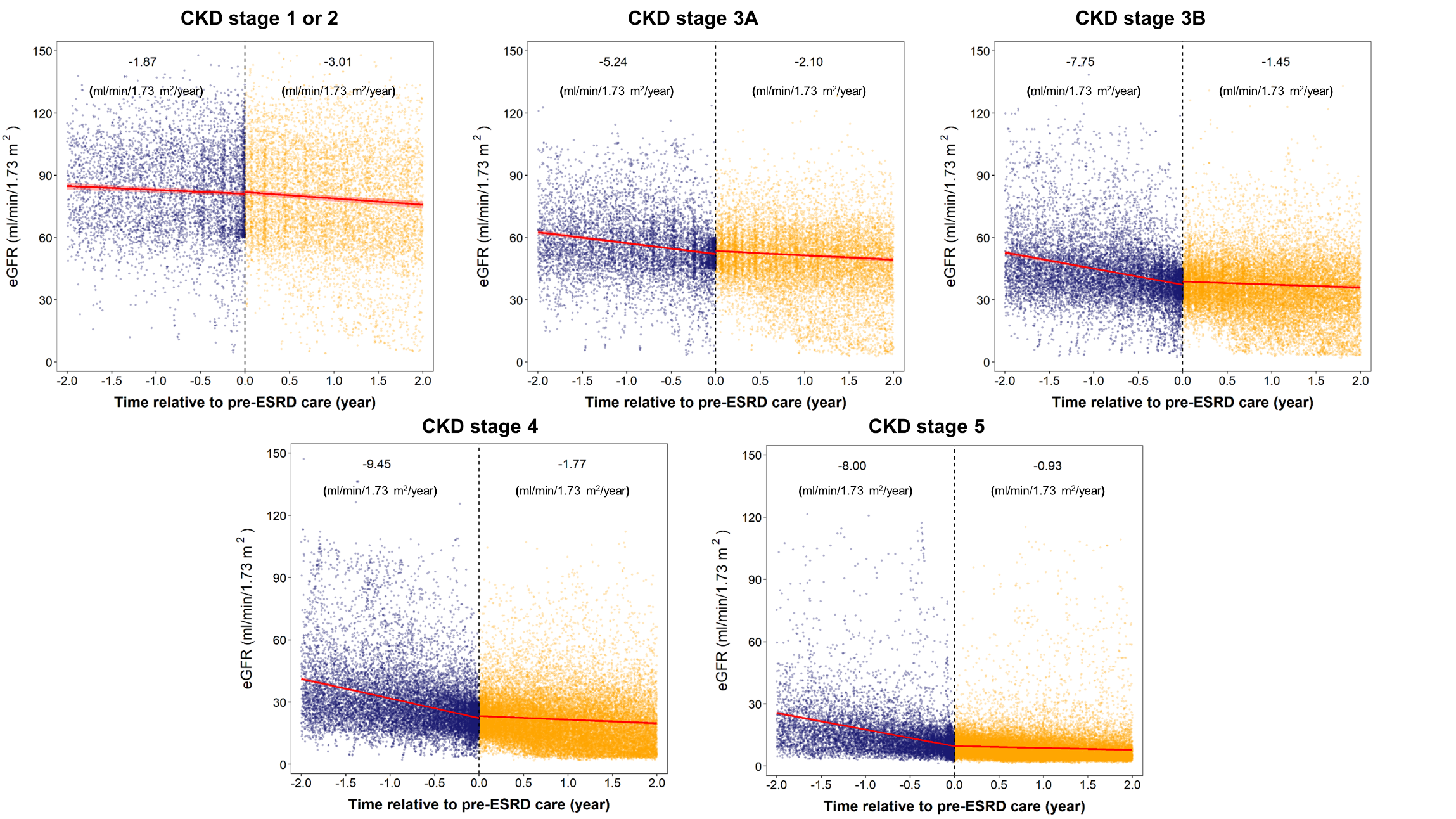

Supplement: Supplementary file 1 — Supplementary Informations. [file 41598_2021_93254_MOESM1_ESM.docx]
